# Phosphorus management in Europe in a changing world

**DOI:** 10.1007/s13280-014-0613-9

**Published:** 2015-02-15

**Authors:** Oscar F. Schoumans, Fayçal Bouraoui, Christian Kabbe, Oene Oenema, Kimo C. van Dijk

**Affiliations:** 1 Alterra Wageningen UR, P.O. Box 47, 6700 AA Wageningen, The Netherlands; 2 Institute for Environment and Sustainability, European Commission - DG Joint Research Centre, Via E. Fermi 2749, 21027 Ispra, VA Italy; 3 Kompetenzzentrum Wasser Berlin gGmbH, Cicerosstrasse 24, 10709 Berlin, Germany; 4 Wageningen University, P.O. Box 47, 6700AA Wageningen, The Netherlands

**Keywords:** Phosphorus, Balance, Resource cycle, Recovery, Waste, Manure, Climate change

## Abstract

Food production in Europe is dependent on imported phosphorus (P) fertilizers, but P use is inefficient and losses to the environment high. Here, we discuss possible solutions by changes in P management. We argue that not only the use of P fertilizers and P additives in feed could be reduced by fine-tuning fertilization and feeding to actual nutrient requirements, but also P from waste has to be completely recovered and recycled in order to close the P balance of Europe regionally and become less dependent on the availability of P-rock reserves. Finally, climate-smart P management measures are needed, to reduce the expected deterioration of surface water quality resulting from climate-change-induced P loss.

## Introduction

Phosphorus (P) is a life-essential irreplaceable element and the Earth’s biomass potential is P limited (Asimov 1959; Smil 2000; Filippelli 2008). As P is often in short supply for optimal plant and animal growth and development, farmers use P fertilizers and add P additives. P also has many industrial applications, including household detergents. However, excessive use of P leads to deterioration of the water quality, eutrophication, and loss of biodiversity.

Essentially, all chemical fertilizer and feed P are derived from phosphate-rich rocks which are located in a few places on Earth and are finite. As Europe has no significant phosphate mines, it is highly dependent on the import of phosphate ore (De Ridder et al. 2012). The current worldwide P reserves are estimated at 67 000 Tg P^1^ and the world mining production in 2013 was 220 Tg P (Survey 2014). About 75 % of the known reserves are located in Morocco (Western Sahara), which is the main exporter of phosphate ore. China and USA also have significant reserves but the phosphate ore is not sold on the global market, which further limits the source of supply for other countries.

Geopolitical changes can cause the price of P fertilizer and hence of food to rise, as happened in 2008 (Cordell and White 2011). Moreover, the challenges related to population increase, increasing urbanization and changes in diets are putting further pressure on the P demand, as agricultural production will have to increase further to meet food demand. Meanwhile, the burgeoning biobased economy and growing demand for bio-energy are increasingly competing with the food production sector for scarce land, fresh water, and other natural resources. These changes may have increasing impacts on climate change, water resource depletion, soil degradation, and air pollution, and could ultimately further constrain food production in the future. These trends and issues can be expected to affect P use efficiency and water quality too.

Recently, it has been proposed to implement a coherent package of nutrient management strategies and measures: the 5R strategy (Withers et al. 2015). This strategy is intended to close the P cycle in Europe. The five Rs are Realign P inputs, Reduce P losses to waters, Recycle P in bio-resources, Recover P from waste, and finally if necessary Redefine our food system. Many Best Management Practices (BMPs) have been developed to increase P use efficiency and reduce P losses (Schoumans et al. 2014a). Here, we build on the 5R strategy and BMPs to discuss the prospects for the two main options for closing the P cycle (reduce the inputs and recover/recycle P from food processing waste, non-food waste, municipal waste, and manure) against the background of the changing world. We start with a brief overview of the P balance in Europe followed by a discussion of the options to close Europe’s P balance and the options for recovering P from waste streams. Although the P losses to surface water are relatively low in terms of the inputs and outputs of the P balance, they still need to be reduced since P losses have a relatively large impact on Europe’s freshwater systems. We therefore close by discussing the possible impact of climate change on P losses to surface waters, in order to evaluate the long-term consequences for the water quality.

## P balance of the European Union (EU-27)

**Fig. 1:**
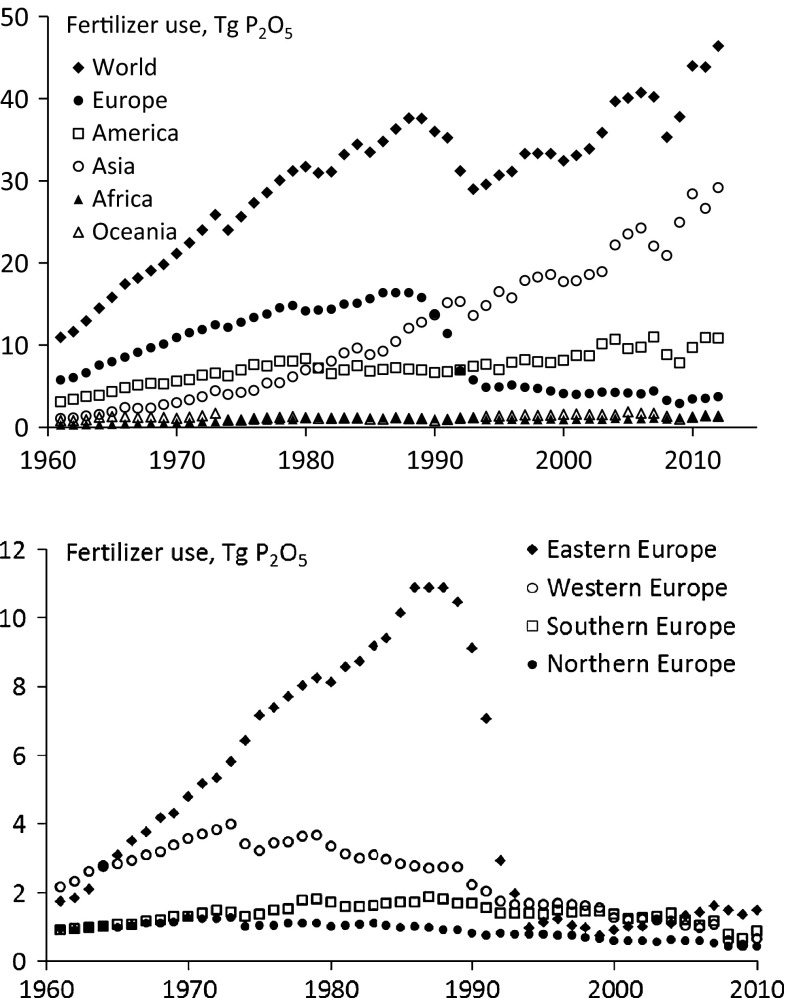
Consumption of phosphate fertilizers in teragram P_2_O_5_ (1 Tg = 10^12^ g = 1 million ton) in the world per continent (*upper*) and per European region (*lower*) during the period 1960–2012 (FAOSTAT 2014). Note the large drop in fertilizer use at the end of the 1980s and beginning of the 1990s in Europe (*upper*) and especially Eastern Europe (*lower*), which resulted from the eastern countries in the former Soviet Union achieving independence and being reassigned (together with their fertilizer use) to Asia rather than Europe

Farmers in Europe began using P fertilizers before farmers in other continents (Fig. 1). Until the 1980s, about half of the annual total P fertilizer consumption in the world was in Europe. Thereafter, consumption in Europe fell sharply in response to the political and economic changes in Eastern Europe and the increasing soil phosphorus status and improved utilization of manure P in the European Union (EU). In Asia, consumption has been increasing rapidly since the early 1980s; that continent now accounts for over half of the world P fertilizer use annually. Consumption in America, Oceania, and Africa is rising slightly.

The picture changes slightly when the consumption of P fertilizers is expressed per unit of agricultural land. Western Europe was the largest consumer during the period 1961–2012 (Table 1), despite the drop in use from the 1970s onwards. During the period 2008–2012, average consumption in Western Europe was at a similar level as the world’s five-year average (Table 1). Mean annual consumption in Europe was 5.2 kg P per ha (equivalent to 12 kg P_2_O_5_ per ha) during 2008–2012. Consumption of P fertilizer in the EU fell appreciably after the price peak in 2008–2009 and has never fully recovered. This contrasts with the rapid recovery in, for example, Asia and America (Fig. 1). Fertilizer use is low in Africa, although most African soils are very low in P, which severely limits crop production (Van der Eijk et al. 2006; Syers et al. 2008b).

**Table 1:** Mean, minimum (min), and maximum (max) values of annual chemical phosphorus (P) fertilizer use per ha of agricultural land per continent and for four regions in Europe (in kg P_2_O_5_ per ha per year) during the periods 1961–2012 and 2008–2012 (based on FAOSTAT 2014)

Region	Period 1961–2012			Period 2008–2012		
	Mean	Min	Max	Mean	Min	Max
World	6	2	10	9	7	10
Africa	1	0	1	1	1	1
Asia	6	3	9	8	6	9
America	8	1	22	17	13	22
Oceania	3	2	4	3	2	3
Europe	13	6	21	7	6	7
Northern Europe	23	11	35	12	11	14
Eastern Europe	8	2	18	4	3	5
Southern Europe	18	10	25	13	10	15
Western Europe	39	8	65	10	8	12

Total P fertilizer consumption in the world quadrupled during the period 1961–2012 (Fig. 1). However, consumption per ha of agricultural land increased less, as the agricultural area increased too, notably in Africa, Asia, and Latin America.

The use of P fertilizer was a major factor contributing to the increase in crop production during the second half of the twentieth century; other important factors were the increased availability of N fertilizers, improved crop varieties and improved crop husbandry. The use of P fertilizer has also contributed to an increase in soil P status in many regions, especially in Western Europe (Reijneveld et al. 2012; Tóth et al. 2014), and thereby has led to a decrease in crop response to P fertilizer application. It is well known that responses in crop yield diminish greatly with an increase in soil P status (SCOPE 1995; Hinsinger 2001; SCOPE 2014), making it uneconomic to apply P fertilizer. This partly explains the large decrease in P fertilizer use in, for example, Western Europe during recent decades.

Few studies have examined the social implications over time of the fate and efficiency of P inputs, and so little data are available on P use efficiency at the level of the entire food chain (e.g., SCOPE 1995; Ma et al. 2013). Table 2 presents the P input–output balance for the EU-27 for 2005 (Van Dijk et al. unpublished data). The main input is chemical fertilizer P, followed by imported food products, animal feed and non-food products. These inputs were used (i) to supply households with food and non-food products, and (ii) to export food and non-food products. Consequently, the apparent efficiency of P use is 57 % (EU food and non-food consumption and P export compared to total P inputs) ([664 + 272 + 544 + 48]/[2659]). However, most food and non-food products to households end up in wastes (691 Gg P) and only part of the P in these wastes is recycled (240 Gg P). If we assume that the output consists solely of the export of products containing P, the P use efficiency falls to 22 % ([544 + 17 + 31]/2659), or to 53 % if we assume that the accumulated P in the root zone of cropland (817 Gg P) is available to growing crops in the long term ([544 + 17 + 31 + 817]/2659). The remaining 47 % (1250 Gg) is considered to be waste streams (mainly organic waste and P losses to surface waters).

**Table 2:** Inputs and outputs of phosphorus (P) (in Gg P) in the European Union (EU-27) in 2005 (Van Dijk et al. unpublished data)

Inputs	Gg P	Outputs	Gg P
Imported fertilizers	1487	Exported food	544
Imported animal feed	417	Exported non-food	17
Imported food products	625	Exported manure	31
Imported non-food products	130	Leaching losses	164
		Manure losses	67
		Food-processing waste	275
		Non-food waste	53
		Municipal waste	691
		Accumulation in agricultural soil	817
Total	2659		2659

Ott and Rechberger (2012) arrived at slightly lower estimates for the P use efficiency in the EU-15 during the period 2000–2010 than we show here for 2005. The main reason is that we have included the new Member States in central Europe, which have higher P use efficiency in crop production than the ‘old’ Member States of the EU-15. However, considerable uncertainty surrounds the data presented in Table 2, which we have compiled from statistics (FAOSTAT, Eurostat), expert interviews, literature, and additional model calculations. The uncertainty in the input and output flows is estimated at roughly between 10 and 50 % for the EU (Ott and Rechberger 2012).

Given the current P inputs (Table 2) and high soil P status (Tóth et al. 2014), the risk that P is limiting crop and animal production in Europe is low, although there is huge spatial variation in P inputs and soil P status across Europe (Csathó and Radimszky 2009). The spatial variation in P inputs is strongly related to animal density and associated P inputs via animal manure. Although the Nitrate Directive (EEC 1991) restricts the annual manure application rate in terms of amount of nitrogen, there is no European policy on P legislation, although some EC Member States have introduced limits to P application (Amery and Schoumans 2014).

## Options to close the P balance in Europe

The legacy of past fertilizer and manure applications in soil P reserves can be used for crop nutrition during subsequent decades (Sattari et al. 2012), though for how long depends on how effectively the accumulated soil P is used. In areas with high soil P status, the P application can be greatly reduced. In the long term, P fertilizer should match P removed in harvested crops, and P import should match P exports plus inevitable P losses. Also, P supplementation of animal feed should be reduced so that the P content matches animal requirements, and efforts have to be made to increase the digestibility of P in feed, especially in the case of feed for monogastric animals (Van Krimpen et al. 2012). Our analysis indicates that European P inputs via chemical fertilizers and animal feed can be reduced but that the European P cycle can be closed only if P from waste streams is recycled back to the soil into the food chain (Table 2). A main challenge is therefore the recovery and subsequent recycling of P from wastes (domestic and industrial) and manure (from intensive livestock farming). Another challenge is to transfer P from regions with excess P (for example, regions with high densities of livestock and/or of people) to regions with the greatest need to replenish depleted soil P pools. Further, decreasing the diffuse pollution of surface waters (P leaching losses from cropland and farmyards) would also contribute to solving the ‘P challenge’.

## P recovery from waste streams

Awareness of the scarcity of P and of the need to close the P cycle has stimulated research on the P recovery from waste streams. As indicated in Table 2, the waste streams in the EU-27 are substantial: the biggest is domestic wastewater, which is mainly collected at waste water treatment plants (WWTPs).

**Fig. 2:**
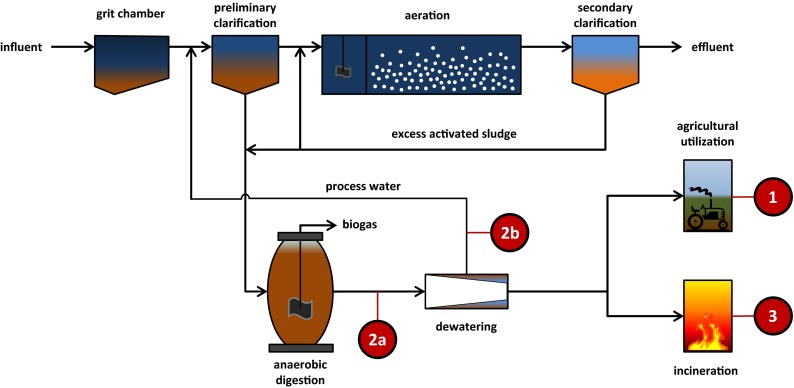
Hotspots for phosphorus (P) recovery from the wastewater stream (in centralized sanitation systems). *Source* Kabbe (2013). *1* Direct sludge application in agriculture; *2a* P recovery from aqueous sludge phase prior to dewatering; *2b* P recovery from sludge liquor after dewatering; *3* P recovery from mono-incineration ash

Figure 2 illustrates the main points for interventions for P recovery in the stream flow of wastewater at a WWTP. Direct application of stabilized and dewatered sewage sludge on arable land is the traditional path to valorize the nutrients from WWTP in agriculture (no. 1 in Fig. 2). But, due to increasing concerns about pollutants, whether known (heavy metals) or unknown (organic contaminants and pathogens), this route is being increasingly questioned by the public and authorities, and some European countries have banned the application of sewage sludge in agriculture (e.g., Switzerland). Therefore, solutions for technically advanced P recovery and recycling have been investigated and in some cases are already being implemented. These alternative routes for nutrient recovery provide safe products or raw materials suitable for reuse in the nutrient cycle of the food chain.

If sludge is incinerated in a mono-incineration plant (no. 3 in Fig. 2), a high P concentrate (P ash) can be produced from waste streams (Adam et al. 2009; Schipper and Korving 2009; Kabbe 2013). The P content in municipal sludge ash ranges between 4 and 13 % (Krüger et al. unpublished data). But, due to low plant availability of the nutrient within the ash, further treatment is needed before it can be fully utilized as a P resource. Due to lack of ash treatment facilities and interim storage capacities, most of the P in the ash is not recovered as easily soluble mineral P. The production of fertilizer from ash via a thermochemical method such as in the AshDec process (Outotec) (Adam et al. 2009; Stemann et al. 2014) involves treating the ash at approximately 1000 °C to remove the heavy metals and to increase the bio-availability of P in the ash. The process produces a magnesium-enriched dicalcium phosphate that can be marketed as chemical fertilizer. An alternative is wet chemical extraction developed in cooperation with companies with experience in treating low-quality phosphate ore (De Ruiter 2014). This process (EcoPhos) would allow the production of phosphates suitable for use in animal feed. Looking at the Mephrec process, thermal valorization and phosphorus recovery in one single step are not mutually exclusive (Scheidig 2009). This technology even offers prospects of recovering precious metals as well as P. The resulting P-rich slag is suitable for fertilizer production and comparable to the well-known Thomas phosphate, a by-product of steel production.

Depending on the wastewater treatment scheme, phosphorus can be recovered from the aqueous phase of the sludge before (no. 2a in Fig. 2) or after the sludge dewatering process (no. 2b). Many mature techniques are available and being developed for sludge treatment (Rulkens 2004; Kabbe 2013; Stemann et al. 2014) and, more specifically, for recovering P from municipal or industrial waste streams (Table 3). Examples of well-known P-recovery techniques are AirPrex, NURESYS, PEARL, PHOSPAQ, the Gifhorn process, and the Stuttgart process (P-REX EU Project, http://www.p-rex.eu/). Phosphorus is often recovered as magnesium ammonium phosphate (struvite), mono ammonium phosphate (MAP), and dicalcium phosphate (DCP) (Greaves et al. 1999; Le Corre et al. 2009; Tan and Lagerkvist 2011; Kabbe 2013).

**Table 3:** Phosphorus (P) recovery in wastewater treatment plants (WWTPs). *Sources* Kabbe (2013) and Stemann et al. (2014)

Process	Scale	Product	Reference
*Sludge/process water*			
AirPrex	Full	Struvite	http://www.nutrientplatform.org/business-cases/bedrijfsnaam/a-tm-z/41-waternet.html
ANPHOS	Full	Struvite	http://www.nutrientplatform.org/business-cases/bedrijfsnaam/a-tm-z/81-aa-en-maas.html
Aarhus Water	Full	Struvite	http://www.phosphorusplatform.eu/images/download/ScopeNewsletter102.pdf
Budenheim	Pilot	DCP	Schnee (2014)
Crystalactor	Full	CaP	http://www.nhm.ac.uk/research-curation/research/projects/phosphate-recovery/Nordwijkerhout/Piekema.pdf
EkoBalans	Pilot	Struvite and NPK	http://www.ekobalans.se/en/kretslopp/i-helsingborgs-stad.html
Fix-Phos	Full	CaP/CSH	Petzet and Cornel (2012)
Gifhorn	Full	Struvite	http://www.asg-gifhorn.de/docs/2007-08-flyer-klaerschlammbehandlungsanlage.pdf
LysoGest	Full	Struvite	http://beta.eliquostulz.com/de/lysogest.html
Nuresys	Full	Struvite	http://www.nuresys.org/content/references
PEARL	Full	Struvite	http://www.ostara.com/sloughUK
Phospaq	Full	Struvite	Abma, W.R., W. Driessen, R. Haarhuis en Van Loosdrecht, M.C.M. (2010) Upgrading of sewage treatment plant by sustainable and cost-effective separate treatment of industrial wastewater. Water Science & Technology, 61(7), pp. 1715–1722
P-RoC	Pilot	CaP/CSH	http://kit-neuland.de/2012/uebersicht/die-phosphor-philosophie/
REPHOS	Full	Struvite	http://www.remondis-aqua.de/aq/industrie/leistungsspektrum/abwasserbehandlung/leistungen/
STRUVIA	Pilot	Struvite	http://phosph-or2014.irstea.fr/wp-content/uploads/2014/01/Nveau_Procede_Recyclage_P-Veolia.pdf
Stuttgart	Demo	Struvite	http://www.recyclingmagazin.de/rm/news_detail.asp?ID=15423&SID=617061192168100100&NS=1
*During or after incineration*			
AshDec (Outotec)	Planned	P mineral	http://www.outotec.com/en/Products--services/Energy/Phosphorus-recovery/
EcoPhos	Demo	DCP	http://www.ecophos.com/#/en/operations/
Mephrec	Planned	P mineral	http://www.nuernberg.de/internet/klaerschlammverwertung/
LeachPhos	Planned	Struvite or DCP	http://www.bsh.ch/de/news/news-detail.aspx?nwsid=9
Thermphos	No longer in operation	P4	Schipper and Korving (2009)

The crystallization of struvite tends to be a favoured approach and is the final step of many P recovery technologies (Table 3), yielding a slow-release fertilizer with excellent plant availability (Römer 2013). By crystallizing struvite directly after the digestion (no. 2a in Fig. 2) within the sludge but prior to dewatering, the efficiency of the sludge dewatering can be increased dramatically, thus substantially reducing operational costs (Heinzmann and Lengemann 2013). These benefits are mainly achieved by lower sludge disposal costs, reduced demand for chemicals (flocculation aid), lower maintenance costs (pipe clogging and abrasion of centrifuges), and a higher overall energy efficiency. An option (LysoGest, PCS GmbH) (Kabbe 2014) to enhance the yield of struvite is being demonstrated at WWTP Lingen in Germany, where thermal hydrolysis is being applied to break down excess activated sludge. As well as improving biogas production in the subsequent digester, the treatment also transforms insoluble or hardly soluble polyphosphates into soluble ortho-phosphate available for struvite crystallization in the sludge water (no. 2b in Fig. 2).

The struvite recovered from the sludge might include more impurities than the material crystallized from the process water after dewatering. But the end products are two registered fertilizers [Berliner Pflanze (BWB), Crystal Green™ (OSTARA)] and every WWTP operator needs to choose the treatment process that best fits expectations, needs and infrastructure. Unfortunately, most of the technologies available are suitable only for WWTPs with biological P removal (Bio-P). If the P is removed by precipitating hardly soluble iron or aluminum salts, direct struvite crystallization is not a sensible option. The P needs to be remobilized, e.g., by lowering the pH, as done in the Stuttgart and Gifhorn processes. A very promising and green alternative is the Budenheim process (Schnee 2014); it applies carbon dioxide under pressure as dissolving agent, which is internally recycled, to reduce the pH and thereafter the released phosphates are precipitated as dicalcium phosphate.

**Fig. 3:**
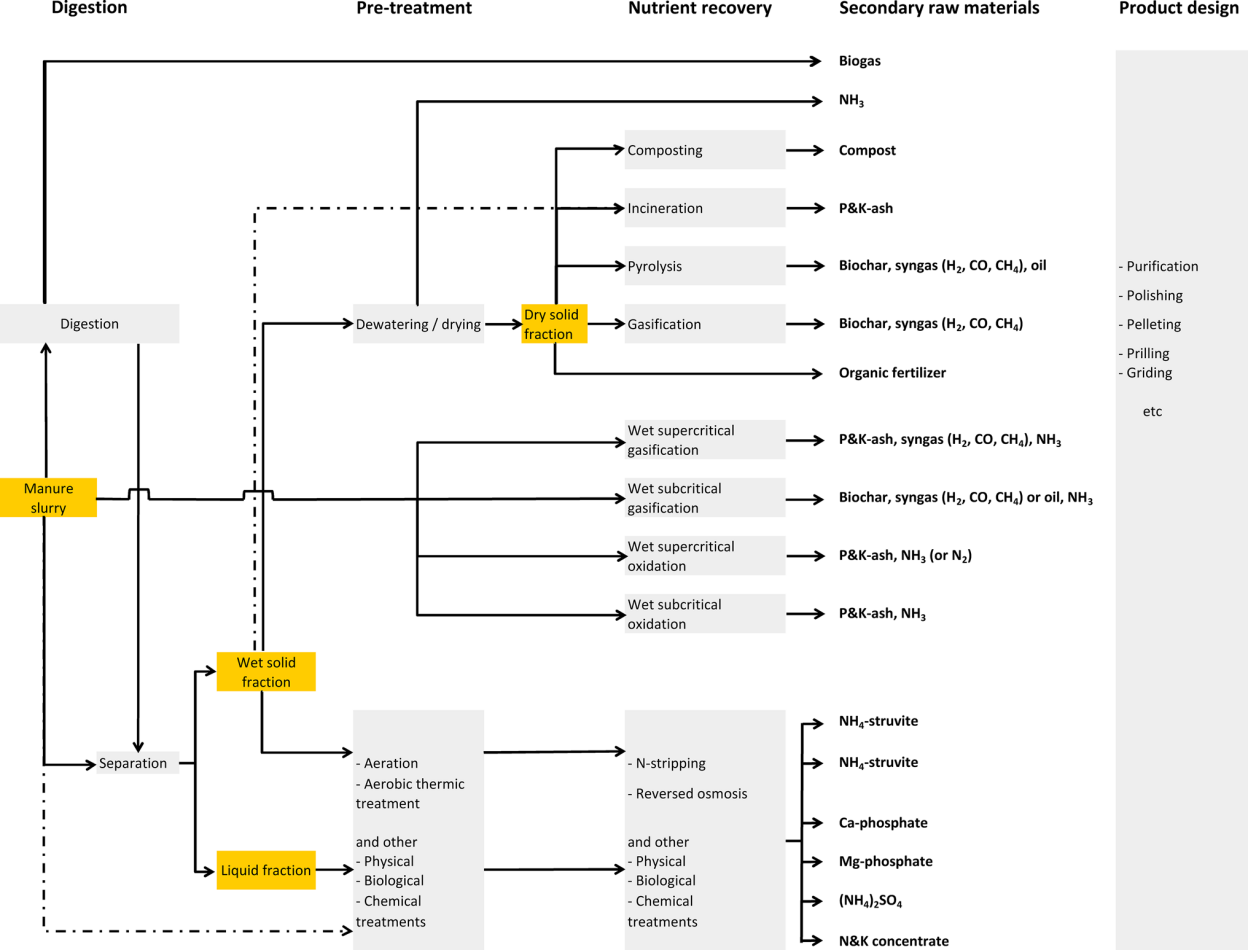
Schematic overview of the main options to recover nutrients from manure in different steps

In regions with intensive livestock farming, P recovery from manure has received much attention. Poultry manure is often incinerated because of its high organic matter content (high energy production) or is dried and exported. Dairy farmers often apply the manure from their cows on their farmland. Consequently, manure processing is mainly focussed on the treatment of pig slurry. Figure 3 gives an overview of the main routes for recovering P from manure slurries.

Nowadays, the most important route of pig slurry treatment is pelleting or composting the dried separated solid manure fraction and exporting the products to other regions. However, the transport costs are still high, and much pig slurry is rejected by crop farms because it has a low N:P ratio which does not match the requirements of most arable crops. This being so, it is more interesting to valorize the components of manure into valuable products.

The solid manure fraction can be used for bio-energy production through incineration and subsequent P recovery from the ash. Both the wet and dried solid fractions of manure can be incinerated depending on the specifications of the incineration plant. The advantage is that manure has a lower iron content than most sewage sludge, and therefore, P ash will contain less iron and is a better as a resource for elementary P production (Schipper and Korving 2009). In addition, also commercial fertilizer industries are able to produce chemical P fertilizers from the P-rich ash.

Another option is to produce a P rich biochar via pyrolysis (Shafizadeh 1982; Wang et al. 2014; Weber et al. 2014) or gasification (Schmieder et al. 2000; Wu 2013). Pyrolysis is a process in which organic matter is heated indirectly to a temperature of 300–550 °C in the absence of oxygen. The organic matter is then converted into a char, pyrolysis oil, water phase, and a gas phase (syngas). The percentage of carbon that was originally present in the manure cake and remains in the char of the pyrolysis process is in the order of 60–70 %. Gasification involves the organic matter being broken down into a P rich biochar and combustible gases at temperatures of usually about 800–1000 °C in a low-oxygen environment. The fraction of the original amount of carbon present in the manure cake that remains in the char is relatively low, in the order of 10 %, resulting in more CO_2_ being produced than in the process of pyrolysis. Pyrolysis and gasification have potential advantages compared to incineration. A main advantage is that the combustible gases of both systems can be converted into electric power more efficiently. Due to the lower temperatures, the plant-availability of the P tends to be higher in pyrolyzed biochar than in untreated P ash (after incineration) or in biochar produced via gasification. The growing interest in producing biochar is in response to the claim that biochar is a soil conditioner and may contribute to fewer emissions of greenhouse gases (Lehmann 2007; Sohi et al. 2009; Spokas and Reicosky 2009).

The complete P recovery from solid manure fractions via incineration, gasification and pyrolysis is attractive because of this manure’s high organic matter content and energetic value. However, there should be also a solution for the disposal of the liquid fraction, since the techniques using whole manure as source are still in development (wet supercritical and subcritical gasification, and wet supercritical and subcritical oxidation; see Fig. 3). In regions with intensive livestock farming there is simply too little land available for applying the liquid fraction (with a high N content, and low organic matter content) and the cost for treatment of the liquid fraction is expensive, resulting in overall treatment costs of manure of €15–30 per ton pig slurry (Schoumans et al. 2010).

More attention has to be drawn to the development of simple precipitation techniques to recover only a part of the P in manure through precipitation of calcium phosphates and/or magnesium phosphates (e.g., struvite) as secondary resource for the fertilizer industries. In principle, the approaches and techniques are the same as for wastewater treatment, but the organic matter content is much higher, which negatively influences the precipitation and crystallization process (Liu et al. 2013; Cerrillo et al. 2014; Zhang et al. 2014). Nevertheless, P recovery via struvite precipitation gives good results in different types of manure (fractions), and high P recoveries (50–90 %) can be obtained from the manure or liquid manure fractions, such as calf manure (Schuiling and Andrade 1999; Siciliano and De Rosa 2014), dairy slurry (Uludag-Demirer et al. 2005; Qureshi et al. 2005, 2008; Zhang et al. 2010; Rico et al. 2011; Huchzermeier and Tao 2012; Uysal and Kuru 2013), pig slurry (Nelson et al. 2000; Suzuki et al. 2006; Shepherd et al. 2009; Jordaan et al. 2010; Capdevielle et al. 2013; Schoumans et al. 2014b) and even poultry manure (Zhang and Lau 2007; Yilmazel and Demirer 2013; Yetilmezsoy et al. 2013). High P recoveries (up to 80 %) from manure or the solid manure fraction can also be obtained using a technique in which acid is applied to release P from the solid fraction into solution, filtering the solution followed by addition of calcium(hydr)oxides to cause calcium phosphates to form (Schoumans et al. 2014b). Since most of the P is in the solid manure fraction, the absolute P recovery is high, but the cost of recovering P is higher than in the case of struvite production (Schoumans et al. 2014b). The main advantages of partial P recovery from manure are that the ratio of N and P in manure becomes more in line with the crop requirements, the organic matter can still be used as a soil conditioner, less chemical P fertilizer is needed and less needs to be spent on exporting manure from areas with a mineral surplus.

## Improving the utilization of P stored in soils

In general, not more than 10–20 % of the applied P is taken up by crops in the first year following application. The remainder is accumulated in the soil, and is a source for crop uptake in the next years (Nuruzzaman et al. 2005; Syers et al. 2008a). When a certain target soil P status has been reached, the P application rate should not exceed the P withdrawal with the harvested crop. In several areas in Europe, soil P status is higher than the target soil P status, especially in regions with intensive livestock production and with vegetable productions. In these regions, P applications can be withheld or the P application rate can be less than the P withdrawal with the harvested crop, because the crop can utilize the residual P of previous applications.

**Fig. 4:**
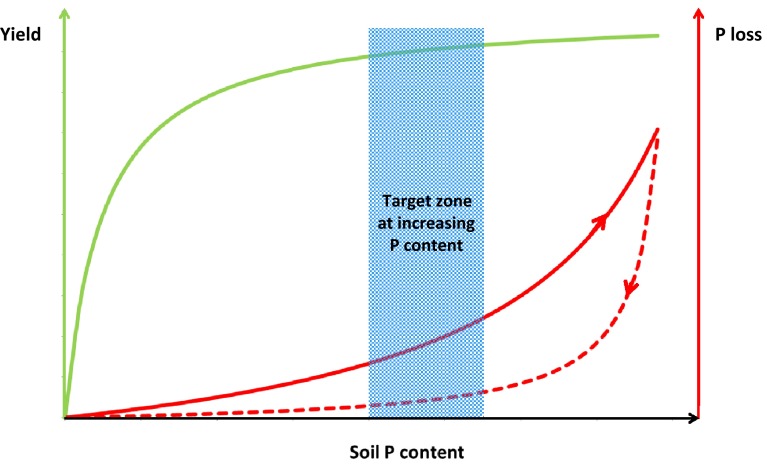
Conceptual representation of yield and phosphorus (P) loss at increasing soil P content (*solid lines*) and associated optimal agro-environmental target zones, according to Tunney (2002). *Dotted line* represent the expected P loss at decreasing soil P content due to mining according to Schoumans and Chardon (2015)

Tunney (2002) and McDowell (2012) argue that there is a small agro-environmental target zone of soil P contents where optimal crop yields and limited P losses can be achieved (Fig. 4). Increasing the soil P status above that target zone would be economically and environmentally unwise. In case the soil P status is above the target range, efforts should be made to decrease the soil P status, through lowering the P application below the level of the P withdrawal with harvested crop, resulting in mining of the soil. It has been argued that the relationship between soil P status and the risk of soil P losses exhibits hysteresis, which means that the relationship between P sorption and P desorption versus the P equilibrium P concentration differs (Barrow 1983) and consequently, loading the soil with P to a high soil P status is associated with a higher risk of P losses than mining the soil P (Schoumans and Chardon 2015) as expressed with the dotted lines in Fig. 4. However, it is unclear whether growing crops will be able to access the residual soil P at a sufficiently high rate to meet the demands at all growing stages under mining conditions. A main challenge will be to manage the residual P in soils and the recycled P fertilizers in such a way that optimal crop growth can be achieved with minimal P losses at reduced soil P levels. If so, the availability of residual P in soil for growing crops and the availability of recycled P fertilizers have to be reconsidered in the fertilizer recommendation schemes for farmers.

## Impact of climate change on P fate and losses

The impact of climate change on the fate of P is difficult to evaluate, due to the multitude of antagonistic and synergistic processes involved in P cycling and transport from source to the fresh waters and finally to the sea. Expected climate change will impact P fate not only directly but also indirectly through effects on the hydrology, on land use and land management—including increased water abstraction, increased fertilization and changes in ecosystems’ composition and functioning. Phosphorus cycling will be affected by changes in temperature and soil moisture regime, and P losses will be affected by changes in the timing and intensity of precipitation.

It is predicted (IPCC 2013) that Europe will experience a significant rise in temperature, while precipitation will increase in Northern Europe and decrease in Southern Europe, with large seasonal variation. Furthermore, it is predicted that extreme climatic events will become more frequent, including heavy precipitation events, heatwaves, extended low-flow periods and droughts. Consequently, climate change will impact the hydrology of most European catchments and will affect the dilution and concentration of phosphorus in surface waters. Recently, the observed increased rain amount during the intense rain events in the Lake Erie has led to an increased phosphorus loading to the lake through runoff, enhancing algal blooms (Sharpley et al. 2015).

Climate change will affect residence and travel time, leading to increased deposition of particulate phosphorus in streams during summer low-flow periods (Whitehead et al. 2009). On the other hand, in Northern Europe, climate change will lead to greater and more frequent flooding, with more interactions between the streams and riparian areas, leading to higher deposition rates of sediment and particulate phosphorus in these areas. Furthermore, extreme high flows and flood events will reduce the retention of phosphorus in streams and will enhance the degradation and transport of particulate phosphorus from stream banks; additional phosphorus loads are expected from more frequent flushing of sewer overflows (Withers and Jarvie 2008).

It is predicted that P loads will increase in Northern Europe, due to increased surface winter runoff and associated sediments (Arheimer et al. 2012), but with large inter-annual variability which might mask the long-term changes expected at the end of the twenty-first century. An increase of total phosphorus load to the Baltic Sea by 15 % is expected in the upcoming 100 years due to climate change (Hägg et al. 2013). An increase of total phosphorus loading by 2050 is predicted for the whole Europe but mostly due to increase discharge from waste water treatment plants and manufacturing (Reder et al. 2013). However, a decrease in river discharge due to climatic change will reduce total P concentration in European streams (Reder et al. 2013). This phenomenon is expected to occur in Ireland, where wetter winters and drier summers with decreased flow are predicted (Jordan et al. 2012).

In Southern Europe, higher evapotranspiration, reduced precipitation and thus a lower water flow are also expected to result in increased concentrations of nutrients, including phosphorus, particularly in river basins dominated by WWTP discharges or by high background P losses. In addition, despite the predicted decrease of annual runoff, very intense rainfall events will be more frequent in Southern Europe, and therefore erosion rates and phosphorus loads will increase (Molina-Navarro et al. 2014).

Higher temperatures are associated with higher mineralization rates (Withers and Jarvie 2008), resulting in soluble phosphorus being more available in the soil profile. The higher water temperature in streams will boost and prolong biological activity, leading to higher primary productivity and higher phosphorus uptake. Similar processes are expected to occur in rivers and lakes. Higher temperatures will increase the release of P from bottom sediment (Feuchtmayr et al. 2009) because the biological activity is higher and continues for longer in the warmer waters, the stratification of organic matter continues also for longer, and higher temperatures increase mineralization of the organic matter in these sediments. Due to prolonged stratification and lack of mixing in lakes, nutrient concentration will decrease at the top of the water column and increase at the lower end of the water column (Jeppesen et al. 2009).

## Discussion

Europe has a long history of net P accumulation due to the large import of P fertilizers (Fig. 1; Table 1) over the last decades. In 2005 a total of 2569 Gg P has been imported to the EU-27, of which 1487 Gg P in P fertilizers and the remainder in food and feed, whereas only 592 Gg P has been exported (Table 2). Currently, overall P exports from the EU-27 are only 22 % of the amount imported, resulting in high P accumulation (in waste and soil; Table 2). Since Europe does not have many phosphate mines and global phosphate rock reserves are limited, there is a need for Europe to close the P cycle.

There is limited scope for reducing the import of P fertilizers by reducing or temporarily abandoning the chemical fertilization of agricultural land with a high P status. That is because such soils are often located in regions with large manure surpluses where the use of P fertilizer is already low. The prospects for reducing the import of P via animal feed seem to be better. Because the feed components are cheap, the P content in the feed exceeds the requirements of the livestock; it seem to be possible to reduce P imported in feed by 20–25 % (Maguire et al. 2005; Van Krimpen et al. 2012; Esmaeilipour et al. 2012) without consequences for the health of the animals. However, such a reduction would have a limited effect on the total P import to Europe.

A substantial part of the net P accumulation in Europe is applied on agricultural land as manure (817 Gg P) and can contribute to the maintenance or improvement of the soil P fertility in areas with a relatively low soil P content. However, P tends to be applied mostly in regions with intensive farming (livestock and/or crop production), where the soils already have high soil P status, thereby increasing P losses to groundwater and surface water (EEA 2012).

The European P cycle could be completely closed and European water quality could be improved if imported chemical P fertilizers were fully replaced by chemical and organic P fertilizers recovered from waste streams (from non-food, food, households and manure; maximum about 1900 Gg P; see Table 2), which can be transported cost-efficiently within Europe (SCOPE 2014). Several techniques are available for recovering P from non-agricultural waste streams (1000 Gg P; Table 2). The incineration of domestic and industrial sludge into P ash and the production of struvite or calcium phosphate from wastewater streams seem to be reliable techniques and are already operating at full scale in North-Western Europe (Table 3). P recovery from manure (potentially about 900 Gg P as amount of P accumulated in soils and amount of manure losses, see Table 2) and conversion into chemical fertilizers is less developed. There are currently no financially attractive options which can compete with the production of fertilizers based phosphate ore, especially if the organic matter (with a low P content) needs to be retained as soil conditioner. However, a number of initiatives and research projects have started studying simple low-tech options for recovering P via different techniques as shown in Fig. 3. The economic success will depend on the overall costs of recovering P and the cost savings for farmers who have to transport the solid P-rich fraction of manure over long distances from regions with intensive livestock farming to areas with low soil P content.

Especially, in the intensive livestock regions the soil P status is higher than the target soil P status and, from an economical and environmental point of view, the P applications can be less than the P withdrawal with the harvested crops. A main challenge will be to manage the residual P in soils and the recycled P fertilizers in such a way that optimal crop growth can be achieved with minimal P losses at reduced soil P levels. If so, the availability of residual P in soil for growing crops and the availability of recycled P fertilizers have to be reconsidered in the fertilizer recommendation schemes.

The P losses from agricultural land to surface water are small compared to the total P balance (164 Gg, which is 6 % of 2659 Gg P; Table 2). However, the consequences for the ecological quality of Europe’s fresh surface water systems are substantial, because diffuse P pollution is seen to be one of the most important factors affecting the eutrophication status of freshwater systems. Losses from agriculture have to be reduced in order to meet the objectives of the Water Framework Directive (EEC 2000). The Nitrate Directive (EEC 1991) regulates the total amount of nitrogen (N) applied on agricultural land, but in most European countries the amount of P applied is not regulated (Amery and Schoumans 2014). In addition, climate change will affect the nutrient losses from land to waters and could undermine the effectiveness of mitigation options. Only a few studies have focussed on the consequences of climate change on diffuse pollution to surface water. The assessment of climate change impact on the surface water quality is complex, because temporal and spatial changes in temperature and precipitation directly influence the crop–soil system and all biological, chemical and physical processes, leading to changes in P concentrations, water flows and pathways. It is expected that due to excess P the surface water quality will decrease, but the causes differ regionally in Europe. In Northern Europe more frequent freezing-thawing of the soil will further accelerate P losses since this will negatively influence resistance again soil erosion and enhance P leaching (Blackwell et al. 2010) in combination with less retention in surface water In Southern Europe the P concentration in surface water will increase due to higher evapotranspiration, reduced precipitation and thus lower flow, and to greater erosion losses due to more frequent rain events. However, it is important to mention that the current large inter-annual variability might mask the long-term changes (Arheimer et al. 2012). Nevertheless, the options to reduce P losses to surface water (Schoumans et al. 2014a) have to be evaluated in terms of Water Framework Directive measures that are ‘climate-change proof’, because the costs (investments and maintenance) of reducing P losses are often high. Such an evaluation should be part of all national action plans to reduce nutrient losses to groundwater and surface water.

For achieving progress toward a circular P economy, active involvement of all actors and stakeholders in the P use chain is required. Several platforms and initiatives at European, regional and national levels have already been established involving all relevant stakeholders. This will contribute to an integration of the options for P recycling and valorization of P waste streams. Involvement of policy makers in these platforms is a prerequisite to ensure that any possible legal barriers are not obstructing possible sustainable solutions. Further, a broad public awareness is needed, as consumers should become more involved in the public debate about minimizing P losses and wastes and the recycling and reuse of wastes.

## Conclusions

The management of P in Europe has to change because global reserves of P are running out, Europe is very dependent on the import of P, and also because climate change will affect Europe’s freshwater quality. The import of P can easily be reduced by (i) applying P fertilizers only where required, (ii) taking into account the amount of P forms in the soil (fast and slow release), and (iii) by reducing the P content in feed to meet the actual dietary requirements of the animals. The biggest reductions can be obtained by reducing or even stopping the import of chemical P fertilizers by recovering P from waste streams and manure and by cost-efficiently transporting the resulting fertilizer products to regions with less P. There are many options to recover P, but many techniques still have to be tested in practice at pilot and full scale, especially those for recovering P from manure. Such effective strategies will increase the efficient use of P and consequently reduce the P losses to the environment, especially when fertilizer P application is fully adjusted to the actual release of P in soils. This P source approach will also help to reduce the rise in P concentrations in surface water that climate change is expected to bring about. Although many measures are available to reduce P losses from agricultural land, they need to be re-evaluated in terms of their climate-proof effectiveness for addressing the objectives of the Water Framework Directive in the long term.

## Footnotes

1 1 Tg = 1000 million kg.
